# Biomechanical study of the sacroiliac fracture fixation with titanium rods and pedicle screws

**DOI:** 10.1590/1413-78522015230300970

**Published:** 2015

**Authors:** Fabrício Hidetoshi Ueno, Marina Justi Pisani, André Nunes Machado, Fábio Lucas Rodrigues, Edison Noburo Fujiki, Luciano Miller Reis Rodrigues

**Affiliations:** 1 Faculdade de Medicina do ABC, Santo André, SP, Brazil

**Keywords:** Pelvic bones, Fracture fixation, Sacroiliac joint

## Abstract

**OBJECTIVES:**

To assess biomechanically different fixations means of the sacroiliac joint with pedicle screws and to compare the traditional head height with reduced ones.

**METHODS:**

We used a polyethylene model representing the pelvic ring and simulated a unilateral sacroiliac dislocation. We set up three different constructions: 1) two screws attached to a rod; 2) two rods connected to two small head screws each; and 3) two rods connected to two average headed screws each. We conducted tests in a biomechanical testing and a mechanized processing laboratory.

**RESULTS:**

Group 1 supported an average maximum load of 99.70 N. Group 2 supported an average maximum load of 362.46 N. Group 3 endured an average maximum load of 404.15 N. In the assembly with one rod, the resistance decreased as compared with the one with two bars: 72.5 % compared to small-headed screws and 75.3 % to the traditional screw.

**CONCLUSION:**

The assembly with a single bar presented inferior results when compared to the double bar assembly. There was no statistical difference in the results between the screws used. *Experimental Study.*

## INTRODUCTION

The pelvic ring fractures are rare and severe. Its incidence is approximately 3% of all fractures and mortality ranges from 6 to 50%.^1^ These injuries are associated with high-energy trauma, remaining a challenge in orthopedic surgery. The treatment of unstable pelvic ring injuries has evolved significantly in recent decades.

There is currently consensus on the surgical treatment for unstable fractures of pelvis^2^ allowing early rehabilitation and decreased morbidity. Yet the patient is not free of problems. In a review article, Tile^2^ reported that 60% of patients with pelvic injuries due to vertical instability have sequelae such as persistent pain, usually in the sacroiliac region, which is often associated with poor reduction of the fracture during surgery. Similarly, Dujardin et al.^3^ showed in their work on pure sacroiliac dislocation that poor functional outcomes are related mainly to the incongruity of this joint postoperatively.

Approaches via anterior, posterior and combined techniques have been described. The posterior approach showed greater stability, especially when associated with vertical injuries.^4^^-^ 7

There are several techniques and implants for fixation and stabilization of the posterior pelvic ring. Among the best known are: screws fixing directly the sacroiliac joint, transiliac plates, sacral bars, percutaneous screw, tension band, and more recently, the use of pedicle screws.^2^^,^ 3 ^,^ 7 ^,^ 8

Biomechanical studies have shown that the fixation by the anterior approach, associated or not with external fixation is insufficient to maintain stability in posterior injuries of the vertical pelvic ring.^9^ There is also the option of posterior approach for the same injury using sacral bars or pedicle screw associated with titanium bars. However, there is no consensus on what is the best way of fixing this joint.^10^^-^ 12

Korovessis et al.^13^ were the first to describe the use of pedicle screws for fixation of unstable fractures of the pelvis associated with dislocation of the sacroiliac joint. They demonstrated the advantage of increasing the use of the interface between the screw head and its multiaxial head and the iliac bone, facilitating the reduction and assembling the fixation.^13^ The same authors have demonstrated biomechanically the increased resistance of pedicle screws when compared with other implants. However, this method requires a good technical and anatomical knowledge of the surgeon to prevent injuries of vascular-nervous structures.^14^^-^ 18 Korovessis et al.^13^ correlated five fixation techniques: one or two cannulated screws in the sacroiliac joint, two anterior superior plates, one pedicle screw in the iliac and one in S1 pedicle, connected by a bar and pedicle screw in each iliac, and one in S1 pedicle associated with a bar. They concluded that the fixation with two cannulated screws had the highest strength and the unilateral pedicle screw had the lower resistance, while the other assemblies presented similar results.

Comstock et al.^12^ in another study tested four methods of posterior fixation: sacroiliac screws, anterior plates, transiliac bars and an association of bars with screws. They achieved the best results with fixation using two iliosacral screws associated with two bars, and the worst results with a single bar.

In another comparison Padalkar et al.^19^ proved that transiliosacral plate is anatomical, radiological and biomechanically as stable as the sacroiliac screws in pelvic fractures with vertical stress, and the low-profile board reduces the risk of prominence and reduces the need for withdrawal of the synthesis material.

The evolution of the sacroiliac fixations showed a significant improvement in the stability of the pelvic ring injuries, but some complications remains, such as the prominence of the implant material due to low muscle coverage of the iliac and sacrum.^19^^,^ 20 Our hypothesis is that, using pedicular screws placed in the iliac bone by a posterior approach, we obtained a satisfactory reduction and sufficient stability for the treatment of unstable pelvic ring fractures by vertical compression. We also believe that the screw with a lower profile will not prejudice the final stability and will decrease the prominence of the material placed in the region. To prove this theory we developed anatomical models of the joint with different assemblies and materials that have been tested in a biomechanical testing laboratory.

The objective of this work is to compare the biomechanical strength of assemblies made with a titanium rod associated with two pedicle screws and two rods with four screws fixed to a pelvis model that simulates injuries by dislocation of the sacroiliac joint. We also aimed to biomechanically evaluate the resistance of a pedicle screw widely used in our midst with another of a lower profile.

## MATERIALS AND METHODs

We performed biomechanical studies in the Biomechanical and Metallographic Testing Laboratory (LEBM) at *Faculdade de Medicina do ABC*. Fifteen specimens were built with characteristics similar to joints that connect the sacral bone with the two iliac bones. This body is formed by two 75mm x 54mm x 32mm polyethylene blocks shaped as a pentagon (ASTM F1717) representing the iliac bones interconnected by a 75mm x 40mm x 64mm polyuretane block representing the sacrum bone. In the simulation of unilateral sacroiliac dislocation, we fixed one of polyethylene blocks (ileum) into the sacrum model with three 6.5 mm cancellous screws, simulating the entire joint, while the other polyethylene block was not fixed, simulating the sacroiliac injury. The study was approved by the Ethics Committee of *Faculdade de Medicina do ABC* under the number 282/2012

In tests, we used 30 mm head length and 5.5 mm diameter polyaxial pedicle screws. We also prepared two types of head profile screw: the commonly used 2 cm high, and the 1.2 cm low profile head. The titanium bars used were 100 mm long and smooth. The fastening of the screws on the rod was made by a pitch system, which is the most widely used on a commercial scale.

**Figure 1.**
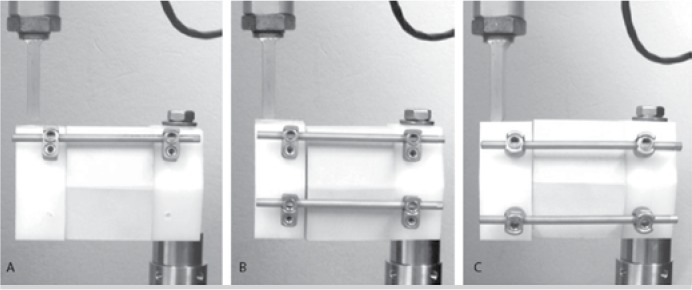
Images of the test bodies mounted in the assay equipment. (A): Group 1 model with one bar and low profile pedicle screws. (B): Group 2 model with two bars and low profile screws. (C): Group 3 model with two bars and normal profile polyaxial screws.

All pedicle screws were fixed in the iliac model. For the study we prepared three groups of five units each: (Figure 1)

- The first group was formed by a bar connecting two low profile polyaxial screws.
- The second group was formed by two bars connecting four low profile polyaxial screws, two screws on each iliac bone.
- In the third group we used four normal profile polyaxial screws interconnected by two bars. This group was named control group, since it is the most common and the most used material in large commercial scale.

**Figure 2.**
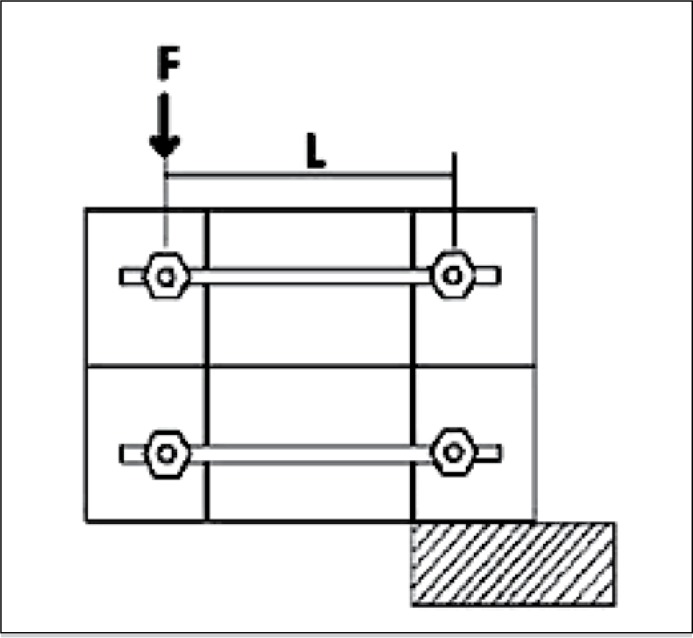
Illustration of load application on the assembly where F is the vertical load applied to one of the blocks representing the iliac bone.

**Figure 3.**
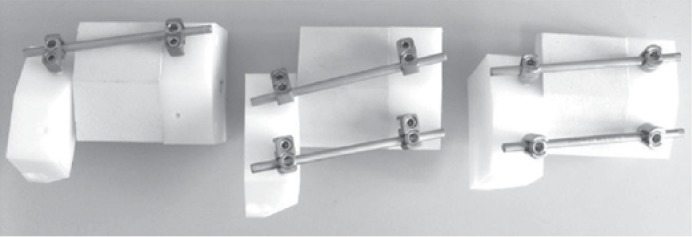
Demonstration of how the test models looked after the tests on the biomechanical tests equipment.

The torque used for inserting the pedicle screws was standardized by using a torque wrench. After placement of the fixation system, these models were tested using a biomechanical testing apparatus. The fixation system was subjected to a unilateral progressive vertical stress at 10 mm per minute until failure of the fixation block. (Figures 2 and 3, Table 1) The assembly produced a curve for each test, as well as a flow load, momentum flow, maximum load and the maximum momentum borne by the specimens.

**Table 1.** Description of assembly equipment and type of test performed.

Assay equipment	EMIC Modelo: DL10000
Equipment capacity	100kN
Load cell capacity	10kN
Validity of the load cell calibration	05/2013
Assay speed	10 mm/min
Room temperature during assay	23 ^o^C
Distance between support and load applicator - L	100.0 mm (± 0.01)
Distance between parallel bars (Except configuration 1)	40.0 mm (± 0.01)
Material of the support blocks	Polyethylene UHMWPE
Material of the intermediate block	Polyurethane with density 40PCF

We used the Mann-Whitney statistical test to verify whether there were differences between the groups and the Kruskal-Wallis test, and whether the double bars group behaves similarly, considering a P value less than 0.05 to be statistically significant. 

## RESULTS

In group 1, fixation with a bar and two low profile pedicle screws, there was maximum average load-bearing of 99.70 N. Group 2, with two bars and four low profile pedicle screws, bore an average maximum load of 362.46 N. Group 3, with two bars and four conventional profile pedicle screws, bore an average maximum load of 404.15 N. (Tables 2-4)

**Table 2.** Test results of Group 1 in relation to slope of the curve, flow load, flow momentum, maximum load and maximum momentum.

Item	Slope of the curve (F/y) (N/mm)	Flow load (N)	Flow momentum *My* (Nm)	Maximum load (N)	Maximum momentum (Nm)
1	20,975.2	30.0	3.00	112.1	11.21
2	20,563.8	52.0	5.20	117.2	11.72
3	14,924.8	45.0	4.50	87.6	8.76
4	16,925.4	48.0	4.80	90.0	9.00
5	18,299.5	42.0	4.20	91.7	9.17
Mean	18,337.72	43.40	4.34	99.70	9.97
Standard Deviation	2,527.67	8.35	0.84	13.80	1.38
U	53.91	0.15	0.012	0.28	0.028

**Table 3.** Test results of Group 2 in relation to slope of the curve, flow load, flow momentum, maximum load and maximum momentum.

Item	Slope of the curve (F/y) (N/mm)	Flow load (N)	Flow momentum *My* (Nm)	Maximum load (N)	Maximum momentum (Nm)
1	43,863.0	73.0	7.30	326.0	32.60
2	34,330.0	102.0	10.20	347.7	34.77
3	34,894.8	55.0	5.50	389.8	38.98
4	33,925.2	75.0	7.50	376.6	37.66
5	31,529.4	65.0	6.50	372.2	37.22
Mean	35,708.49	74.00	7.40	362.46	36.25
Standard Deviation	4,735.59	17.52	1.75	25.44	2.54
U	104.98	0.26	0.021	1.066	0.11

**Table 4.** Test results of Group 3 in relation to slope of the curve, flow load, flow momentum, maximum load and maximum momentum

Item	Slope of the curve (F/y) (N/mm)	Flow load (N)	Flow momentum *My* (Nm)	Maximum load (N)	Maximum momentum (Nm)
1	33,564.7	40.0	4.00	331.1	33.11
2	35,057.1	75.0	7.50	482.9	48.29
3	36,596.3	55.0	5.50	401.0	40.10
4	28,245.3	60.0	6.00	369.5	36.95
5	32,388.3	65.0	6.50	436.3	43.63
Mean	33,170.33	59.00	5.90	404.15	40.42
Standard Deviation	3,174.92	12.94	1.29	58.71	5.87
U	104.98	0.26	0.021	1.066	0.11

Comparing the types of screw profiles mounted with double bar, there was no statistically significant variation in the tests. The assembly with a single bar had a lower resistance as compared to the assembly with double rod. Group 1 showed a decreased resistance of 72.5% as compared to low-profile screws and 75.3% as compared to traditional height screw.

Applying the Kruskal-Wallis test to compare the average results of the three tests we observed in all the variables evaluated (slope of the curve, flow load, flow momentum, maximum load and maximum momentum), we found statistical difference (P <0.05) and, when using the Mann-Whitney test to compare the results obtained from tests 2 and 3 (both with double bar), the difference in all aspects was statistically non significant (P>0.05).

## DISCUSSION

Biomechanical studies have shown that fixation by anterior approach, associated or not with external fixation is insufficient to maintain stability to posterior vertical injuries of the pelvic ring.^9^ These posterior unstable injuries have alternative posterior approaches as well. Several surgical techniques have been described such as the use of sacral bars and, more recently, the use of pedicle screws associated with titanium bars, however these do not come into consensus on what is the best way of fixing the posterior region the pelvic ring.^10^^-^ 12

There is still much controversy about the importance of anatomical reduction in the functional outcome of patients with sacroiliac joint injury. The Dujardin study proved that the alignment of the joint at surgery and the recovery of the patient do not correlate.

The purpose of this study was to compare both types of construction using pedicle screws and titanium rods in unilateral sacroiliac dislocations, as already described in previous work.^13^^,^ 14 We also analyzed the construction with two titanium rods with two types of pedicle screw profiles due to frequent complaints and also often observed during the postoperative returns than traditional screw protrudes after the pedicle fixations. All constructs tested in this study are used in clinical practice.

We found the best result in all aspects with assembly consisting of two traditional profile pedicle screws and two bars, and the worst outcome with only rod. This result is consistent with the literature, illustrated, for example, by the work of Comstock et al.^12^ and Padalkar et al.,^19^ in their biomechanical study, observed that the trans-iliosacral plate is anatomically, biomechanically and radiologically stable in pelvic fractures with vertical stress, just as the sacroiliac screws and that the low-profile plate reduces the risk of prominence and the need for removing synthesis material. We observed the same in our work.

The figures showed that there is no statistical difference between the two types of screws and that their biomechanical characteristics are similar to the traditional screw, which enables its use in clinical practice. The low-profile screw behaves similar to the traditional screw. We aim to implant it in our future patients. Thus, the need for material removal or revision would also be reduced.

Our study suggests placing a second rod. As Comstock et al.^12^ found higher values of resistance using two bars, we arrived at similar values. There is a more than 70% increase of the resistance on the assembly under the worst possible type of stress, the vertical one.

## CONCLUSION

We can conclude that there is no statistical difference between the results in any biomechanical aspect comparing low profile screws and traditional screws. The assembly with a single bar showed poorer results, requiring assemblies with double bars.
